# Sequence-dependent quenching of fluorescein fluorescence on single-stranded and double-stranded DNA†

**DOI:** 10.1039/d2ra00534d

**Published:** 2022-02-16

**Authors:** Jory Lietard, Dominik Ameur, Mark M. Somoza

**Affiliations:** Institute of Inorganic Chemistry, University of Vienna Althanstraße 14 1090 Vienna Austria jory.lietard@univie.ac.at mark.somoza@univie.ac.at; Chair of Food Chemistry and Molecular Sensory Science, Technical University of Munich Lise-Meitner-Straße 34, 85354 Freising Germany; Leibniz-Institute for Food Systems Biology at the Technical University of Munich Lise-Meitner-Straße 34, 85354 Freising Germany

## Abstract

Fluorescein is commonly used to label macromolecules, particularly proteins and nucleic acids, but its fluorescence is known to be strongly dependent on its direct chemical environment. In the case of fluorescein-labeled nucleic acids, nucleobase-specific quenching originating in photoinduced charge transfer interactions results in sequence-dependent chemical environments. The resulting sequence specificity of fluorescent intensities can be used as a proximity detection tool, but can also lead to biases when the abundance of labeled nucleic acids is quantified by fluorescence intensity. Here we comprehensively survey how DNA sequences affect fluorescence intensity by preparing permutational libraries containing all possible 5mer contexts of both single-stranded and double-stranded DNA 3′ or 5′ end labeled with fluorescein (6-carboxyfluorescein, FAM). We observe the expected large quenching of fluorescence with guanine proximity but also find more complex fluorescence intensity changes depending on sequence contexts involving proximity to all four nucleobases. A terminal T (T > A ≈ C ≫ G) in both 3′ and 5′ labeled single strands results in the strongest fluorescence signal and it changes to a terminal C (C ≫ T > A ≫ G) in double-stranded DNA. Therefore, in dsDNA, the terminal G·C base pair largely controls the intensity of fluorescence emission depending on which of these two nucleotides the dye is attached to. Our data confirms the importance of guanine in fluorescence quenching while pointing towards an additional mechanism beyond the redox potential of DNA bases in modulating fluorescein intensity in both single and double stranded DNA. This study should help in designing better nucleic acid probes that can take sequence-dependent quenching effects into account.

## Abstract 2

Fluorescein is commonly used to label macromolecules, particularly proteins and nucleic acids, but its fluorescence is known to be strongly dependent on its direct chemical environment.

## Graphical abstract

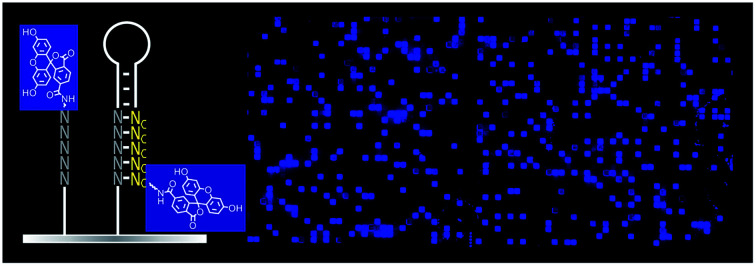

## Introduction

Fluorescein is a ubiquitous dye in labeling, imaging and tracing applications. Historically it is one of the very first fluorophores to enter widespread use thanks to a combination of high molar absorptivity in the visible region (>75 000 M^−1^ cm^−1^), very large quantum yield (*ϕ* = 0.92),^1^ good photostability and solubility in aqueous media. Fluorescein was used in general staining approaches before becoming a macromolecular labeling method, allowing the tracking and quantification of proteins and nucleic acids. Structural derivatives of fluorescein are commonly used as reversible fluorophore tags on nucleoside triphosphates, a pivotal aspect of next-generation sequencing and *in vitro* DNA polymerization.^2–4^ Fluorescein labeling can be conveniently carried out with an isothiocyanate functional group (FITC) or, in DNA and RNA labeling, during solid-phase synthesis by coupling a phosphoramidite version of fluorescein (6-carboxyfluorescein, 6-FAM). The fluorescence properties of fluorescein vary according to changes in the environment and it is most notably sensitive to pH variations, with the highest absorption of 490 nm light at pH > 7 and a progressive decrease in fluorescence response with decreasing pH, which can be understood by the opening of the spirolactone function attached to the xanthene moiety.^5^ Because of its pH-dependent behavior, fluorescein has also found applications in the monitoring of very fine intracellular pH changes.^6^ And while the fluorescence properties of free fluorescein have been extensively studied, much less work has been devoted to studying fluorescein in the context of dye-labeled molecules, specifically how the local chemical environment can affect the fluorescence response. This potential modulation of absorption and emission properties of fluorescence is important to consider and particularly relevant when emission can be correlated to concentration, in nucleic acid quantification and sequencing, but also in more complex photophysical systems based on Förster resonance energy transfer (FRET).^7^

Indeed, fluorescein is a common fluorophore in FRET pairs, but its donor and acceptor properties are sensitive to the nucleic acid environment. The change from single-stranded to double-stranded DNA was found to result in a ∼1/3 decrease in fluorescence intensity^8,9^ and quantum yield is reduced when labeling occurs at the 3′ end.^10–12^ Sequence-dependent fluorescence intensity in oligonucleotides is a well-documented phenomenon affecting most chromophores, although the specific mechanisms are varied. In the case of quenching *via* photoinduced electron transfer from nucleobases, proximity to guanine, as the most oxidizable nucleobase, is the largest contributor. Such quenching is also observed in many commonly used chromophores such as coumarin,^13^ porphyrin^14^ and rhodamine^15^ derivatives, as well as others.^16,17^ Further distal guanine bases also contribute to quenching but to a lesser extent.^18^ Guanosine-mediated fluorescence quenching proceeds *via* photoinduced electron transfer (PET) between the fluorophore and a proximal electron-donor guanine. Since PET efficiency correlates with redox potential at the donor/acceptor level, PET-quenching of fluorescence should follow the order of increasing nucleobase redox potential,^19^ dG ≪ dA < dT ≈ dC, but there is limited data availability on the sequence-specific modulation of fluorescence and any study would likely need to distinguish between 5′ and 3′ labeling, and between single-stranded and double-stranded systems.

We previously investigated the sequence dependence of cyanine dyes in all possible 5mer ss- and dsDNA contexts and revealed how nucleobase identity further away from the dye also affects Cy3/Cy5 fluorescence intensity, as well as that of the structurally similar DyLight DY547 and DyLight DY647.^20–22^ For fluorescein, the modulation of fluorescence by neighboring bases is expected to significantly differ from cyanine dyes, as a π-stacking contribution to fluorescein's interaction with DNA has not been previously documented. Indeed, fluorescence anisotropy measurements show that rotational motion of the fluorescein dye is decoupled from that of the labeled DNA, indicating that fluorescein rotates independently from the nucleic acid molecule.^23^ This effect could be seen as the consequence of the opening of the spirolactone ring at physiological pH, creating not only freedom of rotation about the xanthene–phenolic system but a negatively charged carboxylate as well, which creates a source of electrostatic repulsion with nearby phosphodiester groups,^24^ in clear contrast to positively-charged cyanine fluorophores.

Herein, we explore how the fluorescence properties of fluorescein can be affected by five consecutive DNA nucleotides beginning immediately proximal to the dye, by synthesizing all possible sequence permutations (4^5^, or 1024 unique pentanucleotides) in all terminal labeling conditions, that is 3′, 5′, ssDNA and dsDNA formats. To do so, we synthesized – using nucleic acid photolithography – the DNA oligonucleotides in both 3′ → 5′ and 5′ → 3′ direction with a final, terminal fluorescein coupling using the 6-fluorescein phosphoramidite (6-FAM).^25^ Changes in fluorescence across the surface of the nucleic acid array inform on which nucleobase or ordered combination of nucleobases has the strongest effect on fluorescein emission. As expected, we found that proximal G and G-rich sequences at both the 5′ and 3′ ends of oligonucleotides strongly predict fluorescence quenching. However, the sequence-dependent fluorescence cannot be fully explained by the nucleobase oxidation potential dG ≪ dA < dT ≈ dC. Instead, we measure FAM fluorescence quenching following the order G ≫ C ≈ A ≫ T for 5′ labeled single-stranded DNA, G ≫ C ≈ A > T for 3′ labeled single-stranded DNA, and G ≫ A ≈ T ≫ C for double-stranded DNA. Our data suggest that a redox mechanism alone is insufficient to explain fluorescein fluorescence quenching in DNA. These results should provide comprehensive guidance for better fluorescein nucleic acid probes that can take sequence-specific variations into account.

## Experimental

### Sequence design

**Fig. 1:**
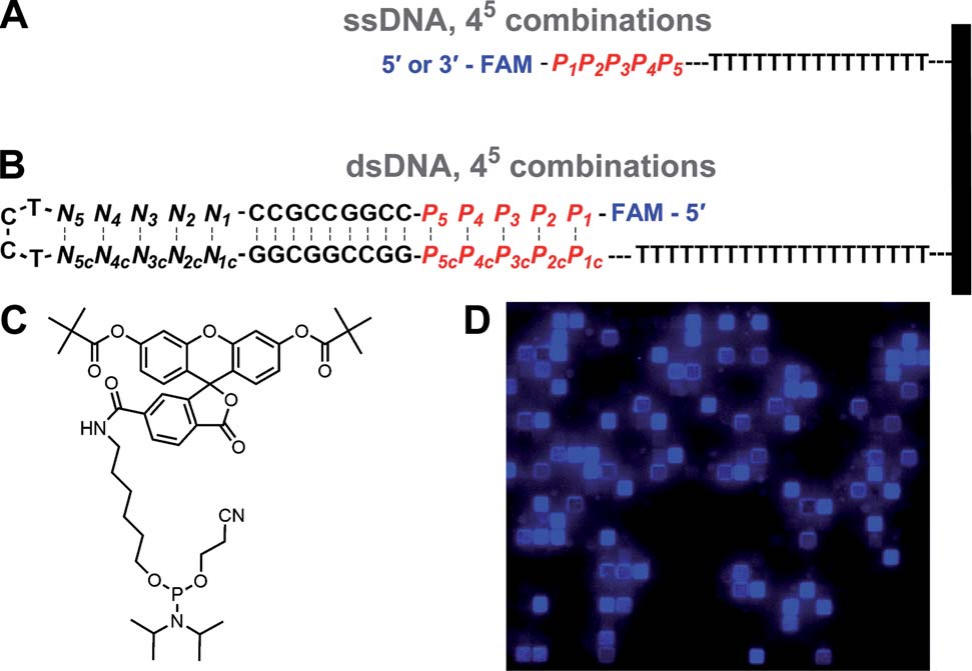
Schematic representation of the (A) single-stranded DNA libraries, and (B) double-stranded DNA libraries with terminal labeling with fluorescein. Each library consists of a complete base permutation set over 5 consecutive nucleotides immediately adjacent to the dye (*P*_1_ to *P*_5_, 4^5^ or 1024 combinations). The identity of the nucleotides *N* in the dsDNA libraries corresponds to the bases absent in the permutation set *P*. For instance, if the *P*_1_·*P*_1c_ base pair is A·T, the *N*_1_·*N*_1c_ base pair is G·C. (C) Structure of the fluorescein phosphoramidite (6-FAM) used to label oligonucleotides. (D) Excerpt of an array scan at 488 nm of a 5′-fluorescein labeled single-stranded DNA library, revealing a large range of fluorescence intensities. Single-stranded oligonucleotide features are randomly distributed throughout the array surface, separated by non-synthesized space serving as background reference. Excerpt is ∼2% of the total synthesis area.

A complete series of base permutations spanning 5 consecutive nucleotides was generated, producing 1024 (4^5^) unique combinations which were installed immediately adjacent to the fluorescein at either the 5′ or 3′ end of oligonucleotides (Fig. 1). The rest of the oligonucleotides were composed of a simple dT_15_ linker to the glass slide surface. The single-stranded DNA library was thus in the form: 5′-FAM-*P*_1_*P*_2_*P*_3_*P*_4_*P*_5_-TTTTTTTTTTTTTTTTTT-(slide) or 3′-FAM-*P*_1_*P*_2_*P*_3_*P*_4_*P*_5_-TTTTTTTTTTTTTTTTTT-(slide). The double-stranded DNA library design was based on our previous work on Cy3 and Cy5 sequence-dependence in ds DNA,^22^ and consisted of a hairpin-forming 36 nt strand with a permuted 5 bp long section at the 5′ end. Close to the hairpin loop (TCCT), a 5 bp-long section is assembled with nucleotides absent from the permutated area, so that each hairpin sequence ultimately contains the same amount of A, C, G and T bases and thus all have approximately equal melting temperatures. An additional core section of GC-rich base pairs in the hairpin ensures a high melting temperature. All hairpins are synthesized on a 20 nt long dT linker. In linear format, the self-annealing structures can be written as 5′-FAM-*P*_1_*P*_2_*P*_3_*P*_4_*P*_5_-CCGGCCGCC-*N*_1_*N*_2_*N*_3_*N*_4_*N*_5_-TCCT-*N*_5c_*N*_4c_*N*_3c_*N*_2c_*N*_1c_-GGCGGCCGG-*P*_5c_*P*_4c_*P*_3c_*P*_2c_*P*_1c_-TTTTTTTTTTTTTTTTTTTTTTT-(slide), where *P*_*i*_ refers to the permutations, *P*_*i*c_ to their complements, and *N*_*i*_ and *N*_*i*c_ to the complementary section used to homogenize nucleobase content while being too distal from the fluorescein to affect sequence-dependent fluorescence. Thus, each G·C base pair in the *P* section has a corresponding A·C base pair in the *N* section, so that the stem of each hairpin contains 5 G·C and 5 A·T base pairs, regardless of permutation.

### Microarray synthesis

Microarray synthesis of DNA oligonucleotides by photolithography proceeded according to procedures described elsewhere and which have incorporated the most recent technical improvements we have brought to the methodology.^25–30,50^ Briefly, Maskless Array Synthesis (MAS) proceeds with 365 nm UV light deprotecting the 5′ or 3′-BzNPPOC photosensitive protecting group (for 3′ → 5′ or 5′ → 3′ synthesis directionality, respectively) at selected locations across the surface of a functionalized glass slide (Schott Nexterion D). Selective photodeprotection is achieved by using an optical relay to image a Digital Micromirror Device (DMD, Texas Instruments) onto the surface of a glass slide forming the entrance window to a photochemical reaction chamber. Mirrors tilted to the ON position result in UV light illuminating a micromirror-sized feature (14 × 14 μm) on the glass surface. Photodeprotection is promoted by the use of a 1% solution of imidazole in DMSO as an exposure solvent. Forward and reverse phosphoramidites (Orgentis) were exposed for 3 J cm^−2^ and coupled for 15 s (forward) and 60 s (reverse). To account for the lower coupling efficiency of G, its coupling time was set to 30 s (forward) and 120 s (reverse), so that all phosphoramidites would achieve a nucleobase-independent coupling efficiency >99%. The fluorescein phosphoramidite (LinkTech) was coupled twice consecutively for 5 min each time at 50 mM concentration in acetonitrile (*vs.* 30 mM for the DNA phosphoramidites). The DNA synthesis reagents were pumped to the synthesis surface with an Expedite 8909 automated synthesizer (PerSeptive Biosystems) using the BzNPPOC phosphoramidites and the exposure solvent but otherwise using the reagents and solvents conventionally used in phosphoramidite chemistry. After each coupling, an additional coupling with a standard DMTr-dT phosphoramidite served to cap BzNPPOC phosphoramidite coupling failures since no reagent in nucleic acid photolithography can unblock a 4,4′-dimethoxytrityl (DMTr) group. This ensures that fluorescein labeling can only occur on full-length oligonucleotides, and together with the high and consistent coupling efficiency, also results in a highly uniform surface density of labeled sequences. After synthesis, the microarrays were washed in acetonitrile for 2 hours at room temperature (rt) to remove unbound fluorescein. DNA arrays were then deprotected in 1 : 1 ethylenediamine/ethanol for 2 hours at rt (12 h for 5′ → 3′ synthesized DNA), washed with distilled water twice and then with 50 mM phosphate buffer at pH 7.6 (PBS) before being spun dry. The dsDNA libraries were self-annealed by heating the microarray in PBS buffer at 50 °C and slowly cooling it down to rt. After 30 min, the array was briefly washed in 1× sodium citrate buffer then spun dry.

### Data extraction and analysis

After drying, the microarrays were scanned in a GenePix 4400A scanner at 2.5 μm resolution with a PMT voltage set at 440 V for both ss and dsDNA libraries. Fluorescein was excited at 488 nm using a built-in solid-state laser and fluorescein emission was collected through a 525 nm bandpass filter. Fluorescence intensity data was extracted from the scan image using NimbleScan 2.1 software (NimbleGen) and data processing was carried out using Excel. The fluorescence intensity values were calculated as an average of all 7 replicates of each sequence randomly distributed across the surface of the same microarray. The intensities were then corrected for background fluorescence for each permutation by subtracting the fluorescence of the corresponding non-labeled oligonucleotide sequence. Error was calculated as standard error the mean. The reported data is an average over three independent replicates. The consensus sequences were obtained by first ranking the fluorescence intensity of the 1024 sequences and dividing the range into 8 bins of equal intensity span. The sequences in each bin were fed into a sequence logo generator (Weblogo, http://weblogo.berkeley.edu/)^31^ and the corresponding consensus sequences were arranged together from high to low fluorescence to illustrate the changes in the sequence dependence of the fluorescence properties of fluorescein. The fluorescence intensity data for all sequences is available in spreadsheet formal as ESI.†

## Results and discussion

The parallel synthesis of all 1024 combinations on the same surface is the ideal approach to study sequence effects on fluorescence intensity in a systematic and reproducible manner.^20–22^ Coupling efficiency is very high (>99%) across all four DNA phosphoramidites and is independent of the identity of previously incorporated nucleotides, as verified *via* sequencing.^29^ This means that terminal labeling should be equally efficient for all sequence combinations and, thanks to efficient capping chemistry,^32^ labeling is prevented on sequences with failed couplings. The effect of distance to the surface on the fluorescent intensity of dyes is well documented^33^ but our sequence design ensures that fluorescein is spaced away from the glass surface by the exact same distance for all combinations (Fig. 1A and B). In addition, since all sequences are synthesized in parallel, synthesis yield and oligonucleotide density are homogenous throughout the entire array surface. The mean distance between DNA molecules on the surface, based on the initial surface density of hydroxyl groups, is also sufficiently large (450 nm) to prevent intermolecular interactions.^34^ Altogether, our fabrication design aims to guarantee a uniform assembly and density distribution of all library elements, allowing relative fluorescence intensity to be compared between sequences. These advantages stand in contrast to solution-based studies, which, in addition to far lower throughput, are likely unable to accurately quantify sequence-dependent relative intensity between different sequences. This is because potential sequence-dependent spectral absorption overlap between fluorescein and DNA prevents an accurate quantitation of DNA concentration, and hence relative fluorescence intensity between the different sequences. Beyond their use in quantifying the sequence dependence of labeling dyes, the very high sequence density along with the highly uniform molecular surface density of *in situ* synthesized nucleic acid arrays has also enabled a variety of ultra-high throughput fluorescence based quantitative assays in molecular biology.^35–42^

**Fig. 2:**
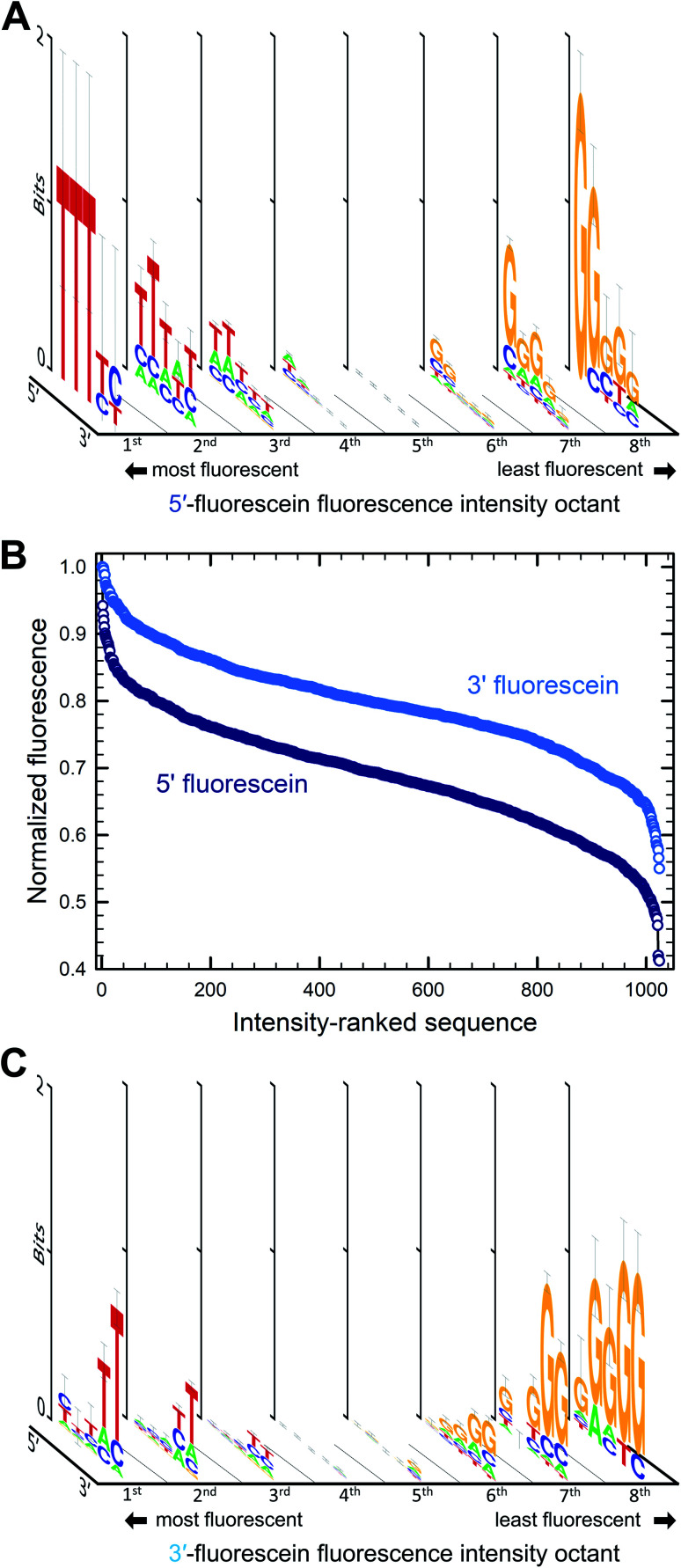
Sequence-dependent variations in the fluorescence intensity of fluorescein-labeled single-stranded oligonucleotides. The relative fluorescence intensity of fluorescein end-labeled 5mers was ranked from most to least intense (highest recorded fluorescence and its corresponding 5mer = 1). For the 5′ fluorescein labeled the intensity falls by 59% and 3′-labeling by 47% (B). The intensity range was divided into 8 equal parts from which consensus sequences were generated and the sequence logos for each octile arranged in descending order of fluorescence intensity (left to right), for 5′ (A) and 3′ fluorescein labeling (C).

We first looked at how the fluorescence intensities of fluorescein are modulated by the sequence context immediately adjacent to the dye, initially in single-stranded format, and then when the dye is attached to double-stranded DNA. We find that sequences interact differently with fluorescein, creating a large range of fluorescence intensities across the 1024 different combinations. In both the 5′- and 3′-labeled oligonucleotide series, the distribution of fluorescence intensities adopt a sigmoidal shape, with up to an almost 55% difference between the brightest and darkest 5′-fluorescein-labeled sequence combinations and a somewhat smaller difference in the case of 3′-fluorescein labeled oligonucleotides, a maximum of 45% quenching relative to the brightest sequence (Fig. 2B). This dynamic range of fluorescence is in line with our previous observations on Cy3 and Cy5 dyes on similarly complex DNA libraries,^20–22^ indicating that the extent of fluorescence quenching in xanthene-like structures is comparable to that in cyanine derivatives. At the top end of fluorescence intensity, we identify the 5mer 5′-TTTTT and 3′-CTTTC (3′-TTTTT being a close third). At the lower end of the fluorescence spectrum, we find 5′-GGGGG and 3′-GGGGC. Clearly, T-proximal single-stranded DNA sequences minimally quench fluorescence while G-rich elements near fluorescein lead to the greatest loss of fluorescence, largely as expected due to the known mechanism of photoinduced electron transfer between the fluorophore and a proximal guanine as electron donor.

To assess the sequence-dependence derived from these very large datasets, we divided the extent of fluorescence intensities into octiles of equal intensity ranges and looked for sequence motifs. Sequence logos were generated for each octile and arranged by intensity (Fig. 2A and C). In both 5′ and 3′ labeling, T-rich sequence combinations populate the high fluorescence intensities while the G-rich counterparts are very likely to be found in low fluorescence data. The top and bottom 1% of fluorescence intensity very clearly show the predominance of T and G nucleotides in the extremes of the intensity range. There is a loss of consensus in the middle range of fluorescence, indicating that the sigmoidal curves can be interpreted as cumulative distribution functions with relative fluorescence as the variable. More intuitively, this pattern originates because most sequences from the full permutational library are composed of a mix of nucleobases associated with both high and low fluorescence, whereas only a few sequences exist that are primarily composed of these same nucleobases, T and G. Unsurprisingly, and corresponding to the electron transfer mechanism, the nucleotide immediately adjacent to the dye is the most important with regards to modulating fluorescence properties, in both 5′ and 3′ labeling and the identity of the nucleobases further away from the terminal nucleotide quickly becomes less relevant.

The sequence-dependence of fluorescein in single-stranded DNA correlates fairly well with our initial observations with terminal nucleotides alone and generally agrees with the expectation that nucleobase redox potential is the most important physicochemical parameter to consider when studying fluorescence quenching in fluorescein. This mechanism, however, would predict that both pyrimidines would have least affected the fluorescence response, while our data demonstrates a strong preference for thymine only. Cytosine-rich combinations (≥4 dC) result in similar fluorescence to dA-rich combinations, in both cases much lower than dT-rich and significantly higher than dG-rich sequence combinations. In terms of nucleobase abundance alone, quenching follows the order dG ≫ dC ≈ dA ≫ dT for 5′ labeling and dG ≫ dC ≈ dA > dT for 3′ labeling, the former reflecting the clear dominance of dT in the set of highly fluorescent 5′-labeled fluorescein DNA conjugates. These patterns follow the dG ≪ dA < dT ≈ dC order expected from their redox potential mostly for dG and its associated quenching.

While the identity of distal nucleotides is less conserved in the brightest and darkest range of fluorescence intensity, they do affect the recorded fluorescence signal. The 5′-TGGGG permutation is amongst the bottom 2% of the fluorescence intensity range and the 5′-GTAAA is part of the first octile of fluorescence. However, a single T or G inserted five nucleotides away from the terminal dye poorly influences quenching, with 5′-GGGGT one of the darkest sequence variant and 5′-TTTTG in the first octile of fluorescence. These observations indicate that on top of the photoinduced electron transfer taking place at the fluorophore–nucleotide level, the neighboring bases can affect fluorescence intensity. Single-stranded DNA is a flexible molecule with a persistence length on the order of a few nanometers^43^—longer than a 5mer—which therefore presupposes that on the length scale of the permuted sequences in our experiments, there exists a partial order and base stacking. Such base stacking in ssDNA has been observed experimentally^44^ and could facilitate charge transport mechanisms between adjacent guanines and through adenine tracts.^45,46^ The redox potential of cytosine and thymine is too large to allow participation in any charge transfer mechanism. Conversely, the flexibility of ssDNA coupled with the very flexible six-carbon linker to the fluorescein should also permit direct contact between the fluorescein and any of the nucleobases of the permuted 5mer. Since guanosine in each of the five positions quenches fluorescein fluorescence, it is clear that some such charge transfer mechanism to distal guanosines is available. Since quenching by distal guanosines is almost entirely absent in the double-stranded DNA data (see below), we can hypothesize that molecular-flexibility-enabled direct contact between fluorescence and guanosine in any of the five positions can result in quenching in ssDNA only.

**Fig. 3:**
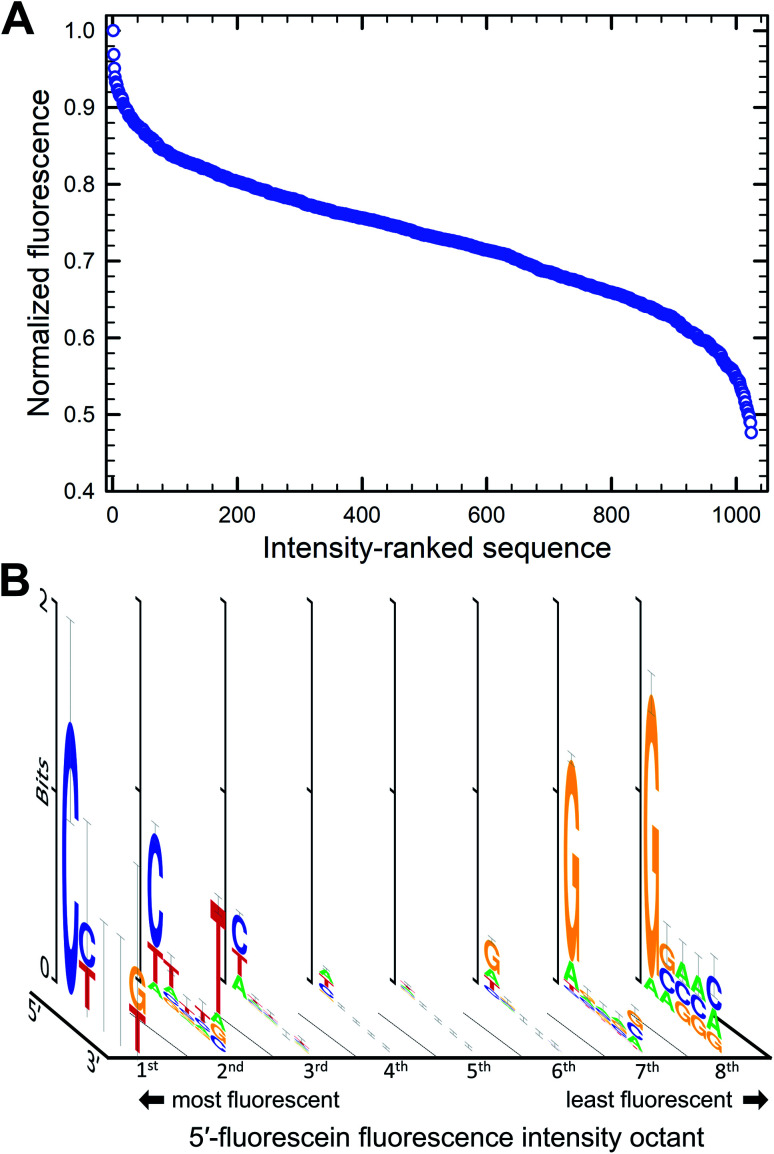
Sequence-dependent variations in the fluorescence intensity of fluorescein-labeled double-stranded DNA. The relative fluorescence intensity of fluorescein end-labeled hairpins was ranked from most to least intense (highest recorded fluorescence and its corresponding 5mer = 1), falling down by a maximum of 52% (A). The intensity range was divided into 8 equal parts from which consensus sequences were generated and the sequence logos for each octile (B) arranged in descending order of fluorescence intensity (left to right).

We next looked at double-stranded DNA with 5′-fluorescein adjacent to the 5-basepair-long permutation region (Fig. 3B). Here too, the intensity of fluorescence varies with sequence, with more than 50% fluctuation between the brightest and darkest sequence combination (Fig. 3). The brightest sequence is 5′-CTACG and the least fluorescent is 5′-GGGCC. As for single-stranded systems, a dG nucleotide next to the dye almost always decreases the fluorescence of fluorescein and can be found in more than 80% of all sequences in the 8^th^ octile of fluorescence. Unlike single-stranded oligonucleotides however, bright sequence combinations frequently present dC at the 5′ end instead of dT (>2/3 of all sequences in the 1^st^ octile of fluorescence). This observation is more in line with the fairly similar redox potential of pyrimidine bases which, based on this metric alone, should indeed predict that dC and dT both do not quench fluorescence intensity. But it is interesting to note that in this context, the nucleotides are base-paired and a dG·dC base pair can drive the fluorescence of the labeled hairpin towards the bright or the dark region depending on which heterocycle is in direct proximity to the dye. The oxidation potential of a G·C base pair was calculated to be lower than the oxidation potential of dG alone,^47,48^ suggesting that photoinduced electron transfer *via* oxidation of the neighboring G base would be more facilitated in base-paired systems which might explain the slightly more dominating presence of G in the most quenching dsDNA combinations. Similarly, the oxidation potential of an A·T base pair was also found to be lower than A or T alone, but an A·T base pair at the very end of a dsDNA molecule is more likely to exist as loose nucleobases (“frayed ends”). The fact that a 5′-C in a hairpin system can be assumed to be correctly base-paired contrary to a 5′-T could by the reason why C appears at the bright end of the intensity spectrum. Furthermore, the importance of the nature of the final 5′ nucleotide suggests that the fluorescein molecule mostly interacts with the closest covalently-bound nucleotide and does not reach over to the complementary base, nor does it appear to intercalate between base pairs either. The identity of the nucleotides further away from the dye does not substantially affect fluorescence intensity, but looking at the ranked list of sequences, the top 5% of fluorescence is very C/T rich, while the bottom 5% is mostly G rich. With pyrimidines consistently found in the top section of the intensity spectrum, it appears that stacking energies, greatest for purines, do not contribute significantly to quenching, even in very rigid double helical structures. The brightest fluorescein-labeled ssDNA is here in the 2^nd^ octile, 20% darker than the top sequence combination.

**Fig. 4:**
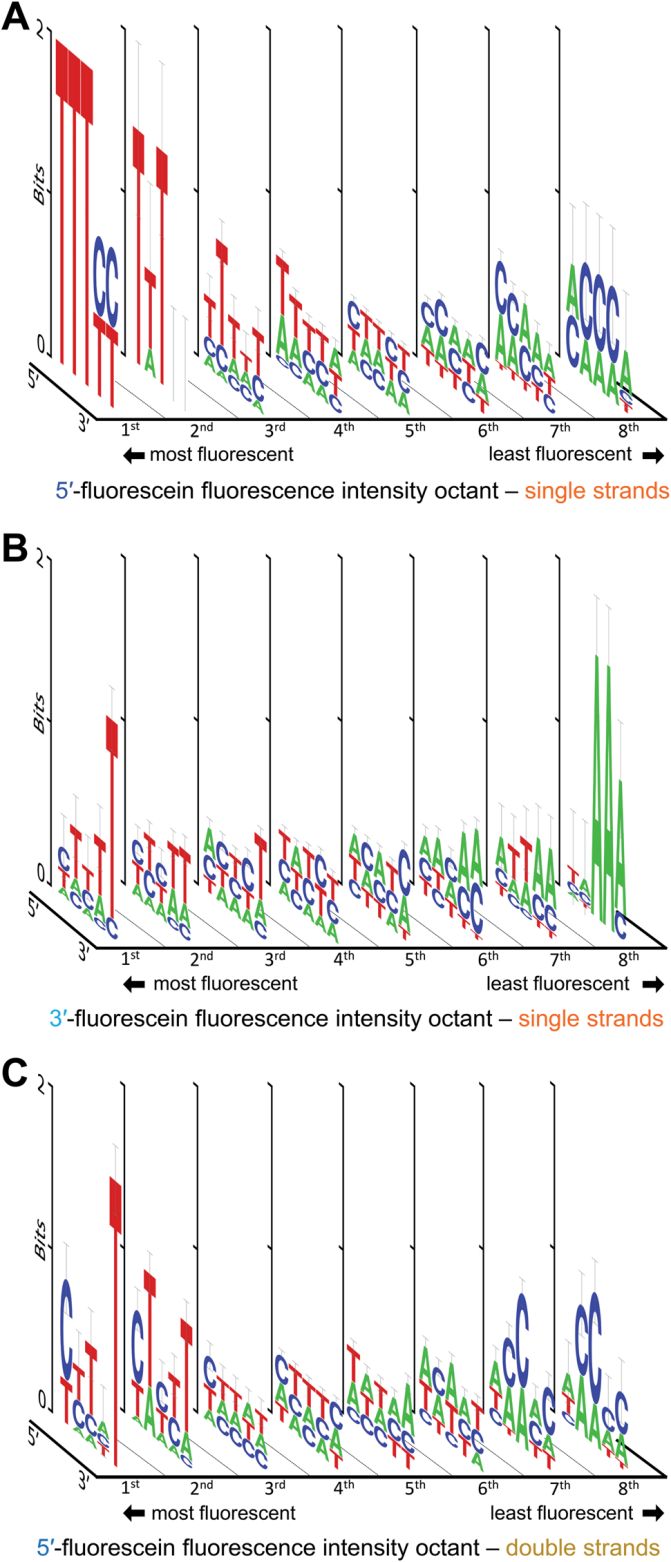
Sequence-dependent variations in the fluorescence intensity of fluorescein-labeled single and double-stranded DNA in the absence of guanine. The intensity ranges for single-stranded 5′-fluorescein (A), 3′-fluorescein (B) and double-stranded 5′-fluorescein-labeled sequences (C) was divided into 8 equal parts from which consensus sequences were generated and the sequence logos for each octile arranged in descending order of fluorescence intensity (left to right).

We also looked at how the sequence context, in the absence of guanine, affects the fluorescence intensity of fluorescein. The results are shown in Fig. 4. With or without G, the strong fluorescence response in single-stranded DNA remain largely dominated by T when in proximity to the dye. Low fluorescence G-free sequences are usually populated with A in 3′-labeled strands (Fig. 4B), which is in contrast with 5′-labeled strands, where low fluorescence is equally distributed between C- and A-rich DNA (Fig. 4A). The fluorescence intensity falls by ∼40% for 5′-fluorescein and by ∼30% for 3′-fluorescein, indicating that some sequences entirely devoid of guanines can still significantly quench fluorescence, with 5′-CACCA and 3′-AAATT producing the weakest fluorescence in all G-free combinations. Even in the absence of guanine, a clear T → C → A transition is difficult to identify when ranking fluorescence intensities from high to low, as the appearance of C in the low fluorescence regime is concomitant with the appearance of A. A-rich sequences can therefore tune the fluorescence properties of fluorescein in single-stranded formats; indeed >50% of all nucleotides in the 5mers that are at least 20% less fluorescent than the brightest sequence combination are composed of A. Since T is prominent in the most fluorescent ssDNA sequences, the T linker to the surface may contribute to higher fluorescence; nevertheless, this would not affect our measured sequence dependence as all ssDNA permutations share this same linker.

In double-stranded DNA, excluding G from the 5mer immediately adjacent to the dye reveals a slightly different picture (Fig. 4C). As was observed in Fig. 3, the final 5′-nucleotide leading to the strongest fluorescence response continues to be C, as opposed to the T seen in single stranded DNA. Interestingly, low fluorescence sequence combinations are populated with C as well, but only in the second and third nucleotide position. Along with A, these sequences at the low end of fluorescein fluorescence produce A/C-rich motifs comparable to those seen in 5′-fluorescein. The fifth nucleotide position furthest from the dye, here appears to prefer a T for strong fluorescence response. Such a clear nucleotide preference at a 5 nt distance from terminal labeling is striking, but has been observed before.^20^ The presence of T at the 3′-end of the permuted region being critical to high fluorescence intensity may be due to the fluorescein dye stacking not on the terminal 5′ base pair, but rather intercalating further down along the double strand, an effect which could not take place in single-stranded oligonucleotides. The intercalation of the xanthene moiety 5 bp downstream of the 5′ end is conceivable given the flexibility of the C6 aliphatic chain linking the dye to the terminal nucleotide. As with ssDNA, the fluorescein in the dsDNA can also interact with the T linker as illustrated in Fig. 1B, but this interaction is shared among all sequences and therefore does not affect the consensus sequence.

**Fig. 5:**
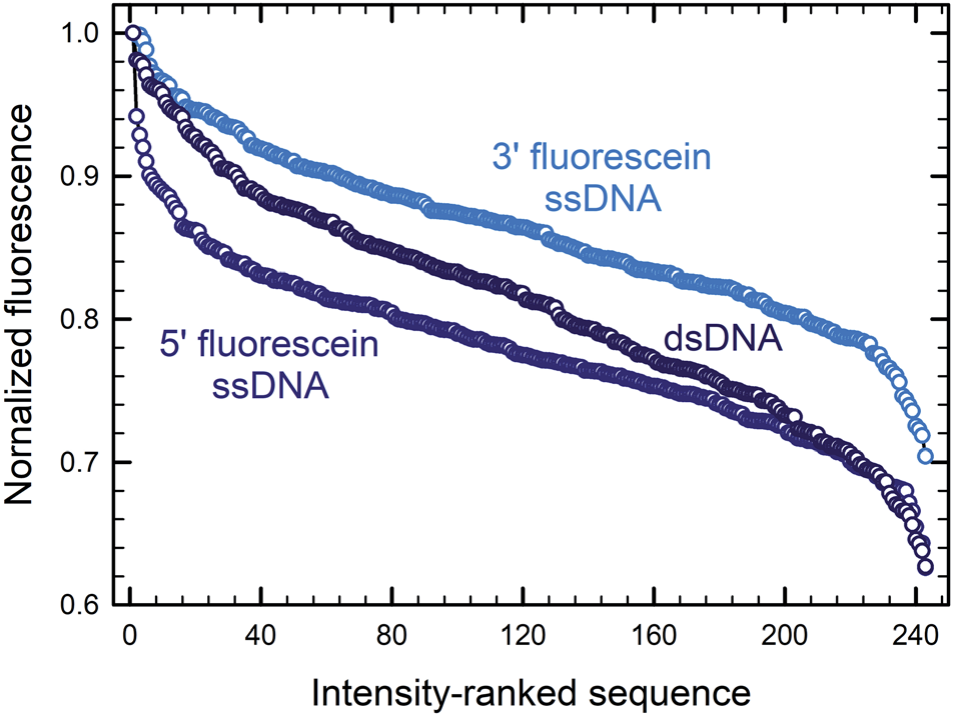
Sequence-dependent variations in the fluorescence intensity of guanine-free 3′ and 5′ fluorescein-labeled ssDNA as well as 5′ fluorescein-labeled dsDNA. The data and normalization are the same as that in Fig. 3B and 4A, but include only the 243 (35) sequences without G in each DNA context. The 5mers are ranked from most to least intense, with fluorescence falling by ∼30% for 3′-fluorescein-labeled ssDNA, and by ∼40% for 5′-fluorescein-labeled ssDNA and dsDNA.

Fig. 5 illustrates how—even in the absence of all guanines—a diminished but still large span of sequence-dependent fluorescence of the fluorescein is retained. The range of intensities, comprising a 30% drop for 3′ FAM ssDNA (*vs.* ∼50% for such sequences including G), and almost 40% for both 5′ FAM ssDNA and 5′ FAM dsDNA (*vs.* ∼60% and ∼50%, respectively, for the equivalent sequences including G). These numbers, along with the discrepancy between the nucleobase redox potentials of A, C and T and their relative prominence in all of the consensus sequences, suggest that one or more additional mechanisms—superimposed on the photoinduced electron transfer mechanism—are needed to fully explain the sequence dependence of fluorescein end labeling in single- and double-stranded DNA. Alternatively, since experimental values for the oxidation potentials have only been determined for free nucleobases in acetonitrile,^13^ significant shifts in more natural contexts cannot be excluded. Within DNA, protonation equilibria,^49^ as well as nucleobase pairing and stacking interactions^48^ may significantly change these potentials, and these changes themselves are likely to be sequence dependent. Even in single-stranded DNA, which is far less structurally defined than double-stranded DNA, base-stacking in ssDNA has been observed experimentally and shown to contribute to its electrostatics and elasticity, two factors which can also contribute to charge transfer efficiency.^44^

## Conclusions

We studied the sequence dependence of end-labeled fluorescein on single and double-stranded DNA by preparing the complete sequence permutation library up to 5 nucleotides adjacent to the dye. We found that the identity of the nucleobase immediately next to fluorescein is the most likely to affect fluorescence intensity and, as expected, fluorescence quenching is largely seen with terminal Gs and G-rich combinations, leading to fluorescence reduction by up to 60% relative to the brightest sequence composition. At this end of the fluorescence intensity spectrum, we noticed that the terminal nucleotide responsible for high fluorescence signals differs between ss and dsDNA, with mostly T as the final nucleotide in ssDNA and mostly C in double-stranded DNA. Distal nucleotides have limited participation in the overall outcome. These results suggest that proximity to guanines, as the most oxidizable nucleobase, is primarily responsible for the modulation of fluorescence intensity in the case of fluorescein, in stark contrast to how cyanine dyes are affected by nucleotide sequence. Beyond guanine, however, the observed sequence dependence of intensity quenching does not correspond to what would be expected based on current experimental values for (isolated) nucleobase redox potentials, suggesting either significant shifts in these values in DNA contexts, or an additional sequence-dependent mechanism that affects fluorescein intensity. The ranking of all sequence permutations by fluorescence can be used as a calibration curve to counter and correct for quenching/dequenching events in labeled probes, for instance in quantitative PCR and more generally in any approach involving FRET and fluorescein. Another type of correction that can easily be implemented is in probe design itself where a terminal G can be accompanied with a T/A-rich segment to partially compensate for G-mediated fluorescence quenching, *i.e.* in the form of GTAAA in ssDNA and GTACT in dsDNA.

## Author contributions

D. A. performed the experiments. J. L. supervised the experiments and analysed the data. J. L. and M. S. conceived the experiments, acquired funding and wrote this manuscript.

## Conflicts of interest

There are no conflicts to declare.

## Supplementary Material

RA-012-D2RA00534D-s001
